# Metabolic Agents that Enhance ATP can Improve Cognitive Functioning: A Review of the Evidence for Glucose, Oxygen, Pyruvate, Creatine, and L-Carnitine 

**DOI:** 10.3390/nu3080735

**Published:** 2011-08-10

**Authors:** Lauren Owen, Sandra I. Sunram-Lea

**Affiliations:** 1 Centre for Human Psychopharmacology, Swinburne University, Melbourne, Victoria 3122, Australia; Email: lowen@groupwise.swin.edu.au; 2 Department of Psychology, Fylde College University of Lancaster, Lancaster LA1 4YW, England, UK

**Keywords:** metabolic agents, glucose, oxygen, pyruvate, creatine, L-carnitine, cognition, ageing

## Abstract

Over the past four or five decades, there has been increasing interest in the neurochemical regulation of cognition. This field received considerable attention in the 1980s, with the identification of possible cognition enhancing agents or “smart drugs”. Even though many of the optimistic claims for some agents have proven premature, evidence suggests that several metabolic agents may prove to be effective in improving and preserving cognitive performance and may lead to better cognitive aging through the lifespan. Aging is characterized by a progressive deterioration in physiological functions and metabolic processes. There are a number of agents with the potential to improve metabolic activity. Research is now beginning to identify these various agents and delineate their potential usefulness for improving cognition in health and disease. This review provides a brief overview of the metabolic agents glucose, oxygen, pyruvate, creatine, and L-carnitine and their beneficial effects on cognitive function. These agents are directly responsible for generating ATP (adenosine triphosphate) the main cellular currency of energy. The brain is the most metabolically active organ in the body and as such is particularly vulnerable to disruption of energy resources. Therefore interventions that sustain adenosine triphosphate (ATP) levels may have importance for improving neuronal dysfunction and loss. Moreover, recently, it has been observed that environmental conditions and diet can affect transgenerational gene expression via epigenetic mechanisms. Metabolic agents might play a role in regulation of nutritional epigenetic effects. In summary, the reviewed metabolic agents represent a promising strategy for improving cognitive function and possibly slowing or preventing cognitive decline.

## 1. Energy Metabolism and Impairments

For all biological and molecular events and for multiple cellular functions, energy is essential. Energy is available in the form of the cellular energy-carrying molecule ATP (adenosine triphosphate), most of which is generated through aerobic cellular respiration of carbohydrate and glucose, the major source of biological free energy in higher organisms. Reduced energy levels threaten cellular homeostasis and integrity. Impaired energy metabolism may trigger pro-apoptotic signaling (programmed cell death), oxidative damage, excitotoxicity and impede mitochondrial DNA repair [1]. These processes can interact and potentiate one another which in turn results in a continuation of energy depletion. At the cellular level, depletion of ATP (the molecular unit of currency of intracellular energy transfer) causes neuron depolarisation leading to failure of membrane ion-transport systems. Calcium influx then leads to the release of neurotransmitters, including high quantities of glutamate, which then activates *N*-methyl-D-aspartate (NMDA) and other excitatory receptors on other neurons. Depolarisation of these neurones causes further exacerbation of calcium influx and further glutamate release. As these processes potentiate one another neuronal insult is amplified. The large calcium influx also activates degenerative enzymes which destroy cell membranes and other neuronal structures. Oxidants such arachidonic acid, and nitric oxide are produced by this process leading to further neuronal damage. Mitochondria provide energy for basic metabolic processes, produce oxidants as inevitable by products, and decay with age impairing cellular metabolism and leading to cellular decline. Mitochondrial membrane potential, respiratory control ratio, and cellular oxygen consumption decline with age and oxidant production increases [2]. Mitochondrial decay may be the principal underlying event in aging [3,4,5]. During normal aging neuronal cell injury and death are accelerated and lead to region specific brain shrinkage. In brain regions particularly important for the formation of memories and decision making, e.g., the hippocampus and the prefrontal cortex/white matter, shrinkage increases with age [6]. Reduction in the total number of viable cells may lead to an accelerated decline in brain functioning. Furthermore a number of diseases and disorders where cognitive impairments are evident co-present with metabolic impairment and neuronal cell loss such as Parkinson’s disease, Huntingtons disease, Alzheimer’s disease and amyotrophic lateral sclerosis [7] as well as mental disorders such as depression [8], schizophrenia [9], and bipolar disorder [10]. Consequently, there has been increasing interest in exploring the use of metabolic agents in combating cognitive decline. Interventions which improve mitochondrial function by sustaining adenosine triphosphate (ATP) levels may have substantial importance for improving neuronal dysfunction and loss. Therefore regimes that buffer intracellular energy levels may impede the progression of neurodegeneration. In the following sections we will provide a brief overview of the metabolic agents glucose, oxygen, pyruvate, creatine, and L-carnitine and their beneficial effects on cognitive function.

## 2. Metabolic Agents

### 2.1. Glucose and Oxygen

All living cells store useful energy in the compound ATP. Glucose is one of the most abundant sugars in our diet, representing 80% of the final products of carbohydrate digestion. Whilst representing only 2% of the total body weight, the brain accounts for 20–30% of total basal metabolism [11] with approximately 120 g of glucose being oxidised by the brain daily [12]. Glucose is either broken down to power the formation of ATP (glycolysis) or stored as long polymers of glucose molecules, e.g., glycogen (glycogenesis). During aerobic (in the presence of oxygen) cellular respiration, the process of glycolysis yields pyruvate. Pyruvate is then transformed to an acetyl group during transition reaction. The acetyl group is used in the Krebs cycle (Krebs cycle or the tricarboxylic acid cycle) and the phase ends with ATP being released in the electron transport chain within the mitochondria. Anaerobic (in the absence of oxygen) respiration/fermentation in human muscle cells is the enzymatic conversion of pyruvate to lactate with lactate dehydrogenase (LDH) which produces ATP in the Embden Meyerhoff pathway [13]. 

Beyond infancy, and under normal conditions, the brains energy requirements are met almost exclusively by the breakdown of glucose. During times of hypoglycaemia other tissues will cease to utilize glucose all together in order to increase glucose availability to the brain [14]. Compared to other organs in the body the brain is particularly vulnerable to small and transient changes in its energy supply. Interrupted delivery leads within seconds to unconsciousness and within minutes may cause irreparable brain damage. Thus the concentration of glucose in the blood plasma is tightly regulated to stay within the normal range of 60 to 90 mg/100 mL for humans. When blood glucose drops below 40 mg/100 mL (hypoglycemic condition) in humans, it can cause discomfort, confusion, coma, convulsions, or even death [15].

Compared with other organs the brain possesses paradoxically limited stores of glycogen, which without replenishment are exhausted in up to 10 min. In nervous tissue, glycogen is stored in astrocytes. Astrocytes participate significantly in brain glucose uptake and metabolism and due to their location and metabolic versatility they may be the “fuel processing plants” within the central nervous system [15]. Glucose deprivation in astroglial cultures results in reduction in the ATP/ADP ratio and membrane depolarization [16] or rapid depletion of glycogen stores [17]. Moreover, cell death results if concentrations of intracellular ATP have fallen within 30 to 48 h to very low levels (lower than 10% of the initial value). Of particular interest to the current review paper is that animal research has shown that inhibition of glycogenolysis in astrocytes can impair memory consolidation [18]. Blood glucose concentrations above the normal range (hyperglycemic condition) can also have damaging physiological effects. Because glucose exerts osmotic pressure in the extracellular fluid, extremely high blood glucose concentrations can cause cellular dehydration. An excessively high level of blood glucose concentration also causes loss of glucose in the urine, which can affect kidney function and deplete the body’s supply of fluids and electrolytes [19].

According to Love and Webb [20], the brain uses approximately twenty percent of the body’s blood and needs twenty-five percent of the body’s oxygen supply to function optimally. Associated measurements of oxygen and glucose levels in blood sampled upon entering and leaving the brain in humans show that almost all the oxygen utilized by the brain can be accounted for by the oxidative metabolism of glucose [21]. While the storage capacity of glucose in the brain is very limited, no storage capacity for oxygen exists in the brain thus cerebral hypoxia (reduced supply of oxygen to the brain) or anoxia (complete lack of oxygen to the brain) leads to almost instantaneous effects on brain function. Since the brain is clearly susceptible to small changes in energy supply, brain function is highly dependent on the availability and metabolism of glucose and oxygen resources.

#### 2.1.1. Glucose and Cognitive Function

The role of glucose in the modulation of cognitive processes is well established. Beneficial effects of glucose administration have been observed across different populations using different experimental paradigms. For example, previous research has demonstrated that glucose administration can enhance learning and memory in healthy young and aged animals and humans (see for example [22,23,24,25,26,27,28]) and may improve several cognitive functions in subjects with severe cognitive pathologies, including individuals with Alzheimer’s disease [29,30] and Down’s syndrome [31]. Facilitation of cognitive performance induced by elevations in plasma glucose levels have also been reported in patients with schizophrenia [32,33]. Furthermore, impairments in certain cognitive skills have been recognized as a possible complication of long-standing non-insulin and insulin dependent diabetes mellitus, and evidence exists that improved glycemic control can ameliorate performance on selective areas of cognition in these populations [34,35]. Although, benefits in cognitive performance have been found in a range of cognitive tasks, converging evidence across populations and methodologies suggest that glucose has most pronounced effects on memory performance [19].

As with many drugs affecting cognitive performance, glucose displays an inverted U-shaped dose-response curve, and its effect is time dependent [36,37]. For young adults 25 g seems to most reliably facilitate cognitive performance however there is some evidence suggesting that the optimal dose may be dependent upon age, inter-individual difference in glucose metabolism and the cognitive domain being assessed [37]. The clearest enhancement effects of increased glucose supply have been observed for verbal declarative memory in a variety of conditions and paradigms [38]. These findings have led to the notion that glucose facilitation may be particularly pronounced in tasks which pertain to the hippocampal formation, which is strongly involved in declarative memory [39]. A further mediating factor for cognitive enhancement by increased glucose availability appears to be the level of task demand. That is, tasks which are more cognitively demanding appear to be more sensitive to the effect of glucose loading [40,41,42]. Also, the data indicates that possible “depletion” of episodic memory capacity and/or glucose resources in the brain due to performing a concomitant cognitive task might be crucial to the demonstration of a glucose facilitation effect. Although the exact underlying mechanisms for this are still unclear, animal research has demonstrated selective reduction in extracellular hippocampal glucose concentrations as a function of the cognitive demand associated with different memory tasks [43]. Moreover, lowered peripheral glucose levels following performance of a cognitively demanding task have been reported [44].

#### 2.1.2. Oxygen and Cognitive Function

It is commonly accepted that oxygen restriction and ischemic deprivation exert marked effects on cognitive function [45]. The global anterograde amnesia resulting from acute hypoxic brain injury due to cardiac or respiratory arrest has also been documented. Clinically, post-hypoxic chronic global amnesia resembles the types of amnesia associated with: bilateral thalamic infarction, Korsakoff’s syndrome, herpes encephalitis and bilateral temporal lobe resection [45]. In addition to non-localised effects, localised lesion patterns can also occur following brain hypoxia. For example, using PET (positron emission tomography), Volpe and Hirst [46] showed that following brain hypoxia, the regional cerebral metabolic rate of glucose consumption was significantly below normal limits in the thalamus and the medial temporal cortex. These data suggests that post-hypoxic amnesia derives from damage to the medial temporal cortex and its thalamic projection areas. However, even transient oxygen deprivation leads to functional but reversible deficits [47,48]. Oxygen administration is capable of reversing the effects of hypoxia with a small therapeutic window, e.g., cognitive deficits from oxygen restriction due to altitude [49], carbon monoxide poisoning [50] and isovolemic anemia [51] can all be reversed by oxygen administration. However impairment effects may be permanent if treatment is not administered in time.

Analogous to the findings that deficit in glucose regulation and utilization, due to age, may contribute to memory impairments in elderly people; age-related cognitive decline has also been attributed to impaired delivery of oxygen through the cerebral vasculature [52,53]. With age, cortical blood supply is reduced to up to 30% [54,55] and additional reduction of regional and total blood flow can be observed in patients with memory impairments [56,57]. Early research by Edwards and Hart examining the effects of hyperbaric oxygen supplementation demonstrated improved cognitive function (short-term memory and visual organization) in elderly outpatients compared to baseline performance. However this study failed to compare with a control group [58]. This study showed significant improvements in mean Wechsler intelligence quotients in elderly participants, who were given 15 daily 2-h sessions of hyperbaric oxygenation. However, other studies showed no reversal of age-related cognitive impairments following normobaric or hypobaric oxygen treatment [59].

Only few studies have investigated the effect of oxygen administration on cognition in normal healthy individuals. In the late 1970s, Walker and Sandman [60] provided indirect evidence that relatively small fluctuations in cerebral oxygen delivery can influence cognitive performance. They investigated the effect of tachistoscopic digit presentation during the various cycles of cardiac output on stimulus processing and EEG parameters. Their results showed that stimuli presented during the P wave (immediately prior to systolic contraction of the heart) were perceived more accurately than those during the T wave (at the end of the contraction). More recently a series of studies investigating the effects of oxygen administration in young healthy adults showed that it led to improved long term memory and reaction times compared to a control group (normal air-breathing) [61,62,63]. Furthermore, similar to glucose facilitation, oxygen administration appears to facilitate cognition most effectively for tasks with a higher cognitive load [61,62]. A further study also examined heart rate during cognitive testing with oxygen *versus* air-breathing controls. Compared to baseline, heart rate was significantly elevated during cognitive testing tasks in both the air and oxygen groups. In the oxygen group, significant correlations were found between changes in oxygen saturation and cognitive performance. In the air group, greater changes in heart rate were associated with more improved cognitive performance [64]. These findings suggest that during times of cognitive demand availability of metabolic resources impact on cognitive functioning. A more recent study has further demonstrated the importance of metabolic resources during cognitive demand by manipulating level of cognitive demand during oxygen administration. In this study oxygen administration of 40% *versus* 21% was examined during completion of an addition task with three levels of difficulty. It was observed that 40% oxygen improved accuracy scores across the task compared to the 21% oxygen dose, with the difference in accuracy rate increasing between the two dosages as the task difficulty level increased [65]. While cognitive demand is clearly a moderating factor for cognitive enhancement by oxygen, enhancement has been observed on several cognitive domains for example oxygen supplementation has been shown to improve everyday memory tasks such as memory for shopping lists and putting names to faces when participants received 100% oxygen compared with air-breathing controls [66]. 

The dose response for oxygen administration on performance appears to follow the inverted U-shaped function which has also been observed to describe the relationship between level of arousal and performance on certain tasks [67] with the most effective doses being one and three minutes for immediate and delayed word recall, and 30 s for tests of attention while continuous oxygen breathing for longer than 10 min leads to decline in performance [61]. The window for cognitive improvement through oxygen administration therefore appears to be quite brief, with research demonstrating that administration of oxygen increases blood oxygen levels for only 4–5 min [61]. In summary, these findings indicate that small increases in the availability of the metabolic substrate oxygen may improve cognitive function.

### 2.2. Pyruvate

Neuronal cell death resulting from hypoglycemia and hypoxia is the result of a series of events triggered by reduced energy availability, and the normalization of blood glucose and oxygen levels does not necessarily block or reverse this cell death process once it has begun. During times of low availability of glucose and oxygen the brain utilizes other, less efficient energy sources that can be produced aerobically. Pyruvate is the end product of glycolysis, which is converted into acetyl CoA that enters the Krebs cycle when there is sufficient oxygen available. When the oxygen is insufficient, pyruvate is broken down anaerobically, creating lactate in humans and animals. Lactate has recently been considered as a central neuroprotective agent [68]. The blood-brain barrier normally transports pyruvate at a rate much slower than glucose, but prior work suggests that significant pyruvate entry to the brain can be achieved by elevating plasma pyruvate concentrations [69].

#### 2.2.1. Pyruvate and Neuroprotection

During pathological insult or general aging the main upstream event most responsible for neuronal cell death is excitotoxicity from glutamate receptor activity [70]. Recent research has shown that cells that would otherwise die after the cascade of excitototic activity could be rescued by providing pyruvate [71]. One recent study assessed the effect of pyruvate administration in rats with hypoglycaemia-induced brain injury. Insulin was used to induce hypoglycaemia and then terminated through (i) glucose administration, and (ii) combined glucose and pyruvate administration. The results of the study showed that in the four brain regions studied (CA1, subiculum, dentate gyrus of the hippocampus, and piriform cortex) combined glucose and pyruvate administration led to a reduction in neuronal death by 70–90%. Increased neuronal survival was also observed when pyruvate delivery was delayed for up to 3 h. The improved neuron survival was accompanied by a sustained improvement in cognitive function as assessed by the Morris water maze [72]. 

Since pyruvate appears to be most beneficial in times of low glucose supply recent research has evaluated the potential usefulness for ethyl pyruvate as a stroke therapy using a rat cerebral ischemia model of middle cerebral artery occlusion. Cerebral ischemia leads to brain injury through a complex series of pathophysiological events leading to neuronal death and subsequent neurological dysfunction. In addition the data suggest that this acute neuronal damage is followed by a second round of neuronal injury, called delayed neuronal death, in the neighbouring areas of the ischemic core [73]. Yu *et al.* [74] found that ethyl pyruvate affords strong protection against the delayed cerebral ischemic injury with significant reduction in infarct volume accompanied by the suppression of the clinical manifestations associated with cerebral ischemia, including motor impairment and neurological deficits.

#### 2.2.2. Pyruvate and Cognitive Function

There is remarkably little research evaluating the effects of pyruvate on cognitive function. However evidence from pre-clinical research has shown that pyruvate infused into the medial septum is capable of reversing the memory-impairing action of morphine [75] and hippocampal infusion has demonstrated reversal of the memory-impairing effects of septal muscimol (a GABA_A_ receptor agonist) [76]. Mild hypoglycemic conditions in rat hippocampal slices interfere with long-term potentiation (LTP) induction [77] and it has been demonstrated that when glucose is unavailable, pyruvate is capable of promoting LTP in CA1 region of rat hippocampal slices [78]. 

There are, as yet, no studies evaluating the effects of pyruvate administration on cognitive function in humans, however pvruvate may be a good candidate for further research in those with energetic depletion and neurodegenerative diseases. Impaired energy metabolism is an early, predominant feature in Alzheimer’s disease and it is believed that impaired cerebral oxidative glucose metabolism is responsible, at least in part, for cognitive impairment in AD. Impairment in glucose utilization in the CNS has been proposed as a possible cause of dementia in Alzheimer’s disease [79]. Research has demonstrated that in both animals and humans increased cerebrospinal pvruvate is a biomarker for AD [80,81] and a significant reduction of pyruvate dehydrogenase (the enzyme responsible for transformation of pyruvate into acetyl-CoA by pyruvate decarboxylation for use in the citric acid cycle) in post mortem AD brain. Since pyruvate administration appears to be quite safe, aside from mild side effects, such as occasional stomach upset and diarrhea, pyruvate therapy might represent an excellent candidate for therapy in disease states with co-morbidity of energetic dysfunction.

### 2.3. Creatine

Creatine (Cr) is a naturally occurring nitrogenous organic acid found in vertebrates. Cr participates in metabolic reactions within cells and eventually is catabolized in the muscles creating creatinine which is then excreted by the kidney in urine. In the averaged sized adult (70 kg), Cr store is approximately 120 g, with the daily turnover of Cr to creatinine being estimated to be about 1.6% of the bodies total Cr [82]. The daily requirement of Cr either through diet or endogenous synthesis is suggested to be approximately 2 g/day [83]. A single 5 g oral dose in healthy adults results in a peak plasma Cr level of approximately 120 mg/L at 1–2 h post-ingestion. The elimination half life of Cr is quite short (just less than 3 h), however elevated plasma levels can be maintained by loading small oral doses every 3–6 h (12–24 g total per day) throughout the day. After a period of loading (1-2 weeks) it is no longer necessary to maintain a consistently high serum level of Cr since there is a maximum tissue storage capacity, with excess being eliminated as waste [84,85,86]. Dietary sources of Cr are fish and red meat [82] therefore Cr levels of vegetarian or vegan individuals tend to be much lower than omnivores. Cr derived from the diet, after passing through the intestinal lumen, enters the bloodstream intact [87] with an average of around 0.25–1 g of Cr per day being obtained from a typical omnivorous diet. 

When Cr is being stored, it is converted to the high energy form of phosphocreatine (PCr) which acts as a high-energy reserve in a coupled reaction in which energy derived from donating phosphate is used to regenerate the compound ATP. In the brain ATP is tightly coupled with Cr and PCr levels within the cell. During times of brain activity brain phospocreatine decrease rapidly in order to maintain constant ATP levels [88,89]. Furthermore, *in vivo* research examining total Cr in the brain, using quantitative localized proton magnetic resonance spectroscopy, have demonstrated that supplementation (2 g per day over 1 month) leads to increases in overall mean concentration of Cr in the brain (8.7%) with region dependent increases [90,91].

#### 2.3.1. Creatine and Neuroprotection

Since Cr is a critical component of maintaining cellular energy homeostasis research has examined its potential neuroprotective effects in various models of neurological disease. Animal research has shown that Cr is particularly important for normal brain development and function. For example depletion of cytosolic brain-type creatine kinase in mice has been shown to result in slower learning of a spatial task and diminished open-field habituation and creatine kinase B-driven energy transfer in the brain is important for mossy fibre field size and determination of seizure susceptibility [92]. Pre-clinical trials have shown that in rats and mice Cr affords significant neuroprotection against ischemic and oxidative insults [93,94,95]. One experiment investigated the possible effect of Cr dietary supplementation on brain tissue damage after experimental traumatic brain injury. Results demonstrated that chronic administration of Cr ameliorated the extent of cortical damage by as much as 36% in mice and 50% in rats. The authors suggested that protection is mediated by Cr-induced maintenance of mitochondrial bioenergetics as they observed that mitochondrial membrane potential was significantly increased, intra-mitochondrial levels of reactive oxygen species and calcium were significantly decreased, and adenosine triphosphate levels were maintained. Induction of mitochondrial permeability transition was significantly inhibited in animals fed Cr [96]. 

Cr may be a good candidate as a neuroprotective agent against acute and delayed neurodegenerative processes. In rodents Cr has been shown to attenuate neurodegenerative symptoms. For example rats administered 3-nitropropionic acid (3NP) display neuropathological and behavioral abnormalities that are analogous to those observed in Huntington’s disease (HD). Rats fed diets containing 1% Cr over an 8 week period showed attenuation of 3NP-induced striatal lesions, striatal atrophy, ventricular enlargement, cognitive deficits, and motor abnormalities on a balance beam task compared to non-Cr supplemented rats. These findings indicate that Cr provides significant protection against neuropathological insult specifically associated with 3NP-induced behavioral and neuropathological abnormalities [97]. An in depth review of the neuroprotective role of Cr is beyond the scope of this review, however a detailed and comprehensive appraisal of the use of creatine in Huntington’s disease, Parkinson’s disease, amyotrophic lateral sclerosis, Alzheimer’s disease as well as spinal cord injury, stroke and epilepsy can be found in “*Creatine and Creatine Kinase in Health and Disease—A Bright Future Ahead?*” [98].

#### 2.3.2. Creatine and Cognitive Function

Despite the obvious impact Cr has on brain development and metabolic actions in the brain there are relatively few studies assessing the effects of Cr on cognitive performance in humans. Clinical trials assessing the effects of Cr on cognitive function can be divided into semi-acute administration protocols (periods of between 5–7 days) and more chronic administration protocols (administration for 6 weeks+). No research has yet examined the acute effects of Cr on cognitive function over the ensuing hours following administration. Furthermore the majority of this research has focused on young healthy adult samples. Cr administration appears to facilitate mood and cognitive function most robustly during times of low energy or fatigue. For example one study examined the effect of 20 g Cr supplementation over 7 days in sleep deprived individuals (24 h sleep deprivation). Individuals who received Cr supplementation demonstrated significantly attenuated performance on a number of tasks including random movement generation, choice reaction time, balance and mood state [99]. In a further study following 36 h sleep deprivation, Cr supplemented individuals also demonstrated improved performance on a random number generation task [100]. One study assessed the effects of 8 g Cr per day for 5 days in healthy individuals and demonstrated attenuation of mental fatigue when subjects repeatedly performed a simple mathematical calculation. In addition, task-evoked increase of cerebral oxygenated haemoglobin was significantly reduced following Cr supplementation, indicating increased oxygen utilization in the brain [101]. A more recent study assessed the impact of a new form of creatine, creatine ethyl ester, over a 2 week period (5 g per day dose compared to dextrose control group) in healthy 18–24 year old participants. The overall findings demonstrated consistent improvements for reaction times across a range of measures as well as improved memory and IQ scores. The strongest improvements appeared to be on tasks that were more cognitively demanding, indicating that creatine supplementation may be particularly useful when performing particularly demanding or complex cognitive tasks [102]. 

Interestingly, to date chronic administration studies appear to show less evidence for improved cognitive performance. For example, following administration of 0.03 g/kg per day for 6 weeks, no effect was observed in healthy young adults on a range of cognitive and psychomotor task (including measures of reaction time, reasoning, information processing and memory) [103]. However, populations who only produce Cr endogenously appear to benefit from additional Cr administration Daily administration of 5 g of Cr (Cr monohydrate) for 6 weeks led to significant positive effects on both working memory (backward digit span) and intellectual efficiency (Raven’s Advanced Progressive Matrices) in a sample of 45 young vegetarian adults [104]. 

Since glucose and oxygen are the primary fuel for brain function it is reasonable that other fuel sources and modes of producing ATP are utilized during times of low energy availability or high energy demands. This preference of energy utilization appears to be reflected in the Cr literature. In that cognition is ameliorated or attenuated specifically during times of metabolic impairment or depletion, either through low creatine availability (vegan and vegetarian samples) or by inducement (sleep deprivation or high cognitive demand). Since elderly populations are generally metabolically impaired and often nutritionally deficient, it seems likely that this population would most benefit from creatine interventions over time. To our knowledge only one study has assessed the impact of Cr supplementation in an elderly human population. McMorris *et al.* administered 20 g of Cr per day for 7 days and found improved performance in a number of cognitive tasks including random number generation, numeric and spatial working memory tasks, and long-term memory tasks [105]. To date there has been no research examining the effects of creatine supplementation in dementia sufferers, however there appears to be some differences in creatine levels in those with genetic risk of developing dementia (apolipoprotein ε4 carriers). Laakso *et al.* demonstrated that compared with non-carriers, the levels of creatine were significantly lower in ε4 carriers. This finding may suggest increased metabolic demands in the brain of the ε4 carriers. They also observed that the levels of creatine correlated significantly with age and performance on the Mini-Mental State Examination test in the ε4 carriers, but not in the non-carriers [106]. 

Although research to date indicates that creatine supplementation might have the potential to ameliorate or ward off cognitive decline in some populations to a certain extend, there is still a great deal of research to be done in this area and a number of questions which remain to be answered. Firstly there has been no research examining whether an acute administration of one single dose of Cr can affect cognitive performance. Secondly since there is some evidence that creatine supplementation improves cognitive function in young, healthy non vegetarian individuals over shorter periods of administration (5 days–2 weeks) but not over longer periods (6 weeks+). Therefore it is possible that adaptation or tolerance on a neuronal metabolic or other physiological level may take place following a certain period of time. Thus further research is required in order to establish optimal dose of Cr and inasmuch optimal dose changes in sub-chronic administration regimes. Moreover, there is a clear lack of investigations into its benefits in populations with mild or marked cognitive impairments. To date only one study has examined the effects in the elderly and no study has been carried out using patients with dementia. Since the evidence suggests that Cr acts to buffer intracellular energy levels and potentially hamper the progression of neurodegenerative processes a more systematic evaluation of Cr mapping cognitive performance over a longer timeframe is required.

### 2.4. L-Carnitine/Acetyl-L-Carnitine

In animals and humans, carnitine is biosynthesized primarily in the liver and kidneys from the amino acids lysine or methionine [107] with Vitamin C (ascorbic acid) being essential to the synthesis of carnitine. In food the highest concentrations of carnitine are found in red meat and dairy products. Other natural sources of carnitine include nuts and seeds, legumes or pulses, vegetables and cereals. Carnitine exists in two stereoisomers: Its biologically active form is L-carnitine, while its enantiomer, D-carnitine, is biologically inactive [108]. Carnitine transports long-chain acyl groups from fatty acids into the mitochondrial matrix, so that they can be broken down through β-oxidation to acetate to obtain usable energy via the citric acid cycle. Under normal nutritional conditions and in healthy persons, L-carnitine availability is not a limiting step in β-oxidation; however L-carnitine is required for mitochondrial long-chain fatty acid oxidation [109], which is a main source of energy during exercise [110]. Furthermore increase in L-carnitine content might increase the rate of fatty acid oxidation, permitting a reduction of glucose utilization, preserving muscle glycogen content, and ensuring maximal rates of oxidative ATP production. In one study L-carnitine improved glucose disposal among 15 patients with type II diabetes and 20 healthy volunteers [111]. Glucose storage increased between both groups and glucose oxidation increased in the diabetic group. Furthermore glucose uptake increased by approximately 8% for both diabetic and non-diabetic groups. 

In neuronal cells, the L-carnitine shuttle mediates translocation of the acetyl moiety from mitochondria into the cytosol and contributes to the synthesis of acetylcholine and of acetylcarnitine [112,113]. The neurobiological effects of acetyl carnitine include modulation of brain energy and phospholipids metabolism, cellular macromolecules (such neurotrophic factors and neurohormones), synaptic morphology, and synaptic transmission of multiple neurotransmitters [114].

#### 2.4.1. L-Carnitine/Acetyl-L-Carnitine and Neuroprotection

A number of studies have examined neuroprotective effects of L-carnitine and acetyl L-carnitine treatment *in vitro*. For example acetyl L-carnitine has been shown to reduce cell mortality in primary cell cultures from rat hippocampal formation and cerebral cortex of 17-day-old rat embryos when cell mortality is induced by 24 h foetal calf serum deprivation. Furthermore, acetyl L-carnitine also protected against exposure to glutamate and kainic. Moreover the neurotoxity induced by *N*-methyl-D-aspartate was attenuated by the acute co-exposure with acetyl L-carnitine. Cell mortality was also investigated in hippocampal cultures chronically treated with β-amyloid fragment in which acetyl L-carnitine also demonstrated neuroprotective activity [115]. Furthermore Ishii and colleagues demonstrated that both acetyl L-carnitine and L-carnitine administration to primary cultured neurons from the cerebral cortex, striatum and thalamus of 18-day-old rat embryos, deprived of serum, promoted neuronal survival (from apoptosis) and mitochondrial activity in a concentration-dependent manner [116].

Pre-clinical *in vivo* research has also demonstrated neuroprotective action of L-carnitine in the rat model of 3-nitropropionic acid (3-NPA)-induced mitochondrial dysfunction. 3-NPA is known to produce decreases in neuronal ATP levels via inhibition of the succinate dehydrogenase at complex II of the mitochondrial electron transport chain. Succinate dehydrogenase is involved in reactions of the Krebs cycle and oxidative phosphorylation, and its inhibition leads to both necrosis and apoptosis. It was observed that pre-treatment with L-carnitine totally prevented the 3-NPA-induced decrease in brain temperature. The authors suggest that the protective effects of L-carnitine against 3-NPA-induced neurotoxicity are achieved via compensatory enhancement of several pathways of mitochondrial energy metabolism [117]. L-carnitine has also been shown to prevent hypoglycemia-induced neuronal damage in the hippocampus, by preserving mitochondrial functions [118]. Acetyl L-carnitine also appears to be effective at promoting neurologic recovery from experimental focal cerebral ischemia in rats [119] and complete, global cerebral ischemia in dogs [120]. 

#### 2.4.2. L-Carnitine/Acetyl-L-Carnitine and Cognition

The majority of research assessing the effects of L-carnitine or acetyl-L-carnitine (acetylated derivative of L-carnitine with improved bioavailability) have focused on its benefits to elderly and populations with dementia. Acetly L-carnitine transverses the blood brain barrier efficiently, and sufficient increase in CSF concentrations following oral and intravenous administration has been observed in patients with severe dementia [121]. In terms of efficacy, a meta-analysis examining the effects of acetyl-L-carnitine in mild cognitive impairment and mild (early) Alzheimer’s disease was conducted [122]. Studies included in the analysis were at least 3 months in duration, with a dosage of between 1.5–3.0 g/day. The results showed beneficial effects on both clinical scales and psychometric tests with improvements after 3 months and even stronger enhancements with longer treatment regimes. In a more recent study the effects of 2 g of L-carnitine daily for 6 days was assessed in centenarians [123] and those treated with L-carnitine demonstrated significant physiological improvements in fat mass, muscle mass, plasma total carnitine and plasma long and short-chain acetylcarnatine. They also showed significantly less mental fatigue and improved cognitive function assessed by the Mini-Mental State Examination (MMSE). 

There are, as yet, no studies examining the effect of L-carnitine or acetyl-L-carnitine on cognitive function in young human populations. Since the action of L-carnitine availability is not a limiting step in β-oxidation any beneficial effects are most likely to be observed in populations with depleted energy resources or under physically fatigued conditions. Therefore the utility of L-Carnitine/acetyl-L-carnitine may be more pronounced in age and degenerative disease, however healthy young populations might benefit in physically demanding and stressful situations. 

## 3. Underlying Mechanisms

Metabolic agents might represent a simple intervention for improving and maintaining optimal cognitive performance across the life-span. In terms of the underlying mechanisms, these agents are directly responsible for generating ATP, the main cellular currency of energy. The brain is the most metabolically active organ in the body and as such is particularly vulnerable to disruption of energy resources. Consequently, the cognition enhancing and neuroprotective effects of these agents might be due to their ability to optimize coordination between ATP consumption and ATP production processes and therefore minimizing metabolite gradients, reducing energy dissipation and providing optimal metabolic energy supplies according to physiological needs. However, the most likely scenario is that the underlying mechanisms are likely to be multifarious and not necessarily restricted to provision of energy necessary for optimal cell function. Recently, it has been observed that diet can affect transgenerational gene expression via “reversible” heritable epigenetic mechanisms. Epigenetic marking on genes can determine whether or not genes are expressed. Among the different mechanisms that could lead to interindividual differences in cognitive ageing and neurodegeneration, the epigenetic regulation of gene expression might be an important contributor. Nutritional, chemical and physical factors have been shown to influence epigenetic events and thereby modifying gene expression profile and disease risk [124]. Although the degree of epigenetic plasticity changes across the life span, epigenetic changes occur at all stages of life and accumulate over time. For example, maximal differences in DNA methylation profile have been observed in aged monozygotic twins with a history of non-shared environments [125,126,127].

Determinants of gene expressions include DNA methylation and intake of meythyl donors (such as choline, methionine, zinc, betaine and folate) might alter gene expression, but also macronutrients and more general agents that alter ATP-dependent complexes [128]. Since it has been shown that diets and/or nutritional compounds which affect energy metabolism can have global epigenetic effects [129], prevention and therapy of cognitive impairments and neurodegenerative processes by diets aimed at optimising bio-availability of metabolic agents present an exciting prospect. Metabolic agents might play a role in regulation of nutritional epigenetic effects, which in turn might account for their benefits on cognition. 

## 4. Summary and Implications

Research is now beginning to identify various agents which may impact on metabolic functioning and delineate their potential usefulness for improving cognition in health and disease. Glucose and oxygen are the primary sources of fuel for the brain and body and thus received the most extensive research attention in terms of their effects on cognitive function. As a result, the optimal dosage, time frame and conditions of administration to reveal facilitation of cognition are becoming better delineated. However the limitations of supplementation of these substrates are also becoming clear and translation into therapeutic interventions is likely to be varied. Oxygen has a very small therapeutic window while glucose supplementation does not represent a viable strategy for neuro-enhancement over any prolonged timeframe since consistently elevated blood glucose leads to insulin resistance. However, therapeutic and nutritional interventions aimed at maintenance of optimal blood glucose regulation are important target candidates. Moreover, other substrates reviewed here include pyruvate, creatine and L-carnitine. These metabolic agents have received less attention in the field of cognition research and subsequently their potential efficacy as cognitive enhancers and neuroprotectants still requires much work. However, the evidence reviewed here provides a promising basis for future research. Mediation of cognitive function by metabolic agents appears to be most robustly observed in those individuals with some metabolic impairment or low energy availability. Future research of metabolic agents for cognitive function could be divided into three main areas: (i) optimising cognitive function in young healthy individuals, (ii) neuroprotection form cognitive decline and (iii) clinical samples with either central or co-morbid metabolic impairment. It is clear that in normal young healthy adults cognition can be altered by even small metabolic fluctuations and that metabolic impairment as a result of aging or disease is associated cognitive decline. Therefore interventions which improve metabolic function or prop up ATP production may have a vast number of implications in health and disease. Finally, the potential role of the reviewed agents as epigenetic modulators clearly merits further research. 

## References

[1] Klein A., Ferrante R., Salomons G.S., Wyss M. (2007). The neuroprotective role of creatine. Creatine and Creatine Kinase in Health and Disease.

[2] Hagen T., Ingersoll R., Wehr C., Lykkesfeldt J., Vinarsky V., Bartholomew J., Song M., Ames B. (1998). Acetyl-L-carnitine fed to old rats partially restores mitochondrial function and ambulatory activity. Proc. Natl. Acad. Sci. USA.

[3] Ames B., Shigenaga M., Hagen T. (1993). Oxidants, antioxidants, and the degenerative diseases of aging. Proc. Natl. Acad. Sci. USA.

[4] Ames B., Shigenaga M., Hagen T. (1995). Mitochondrial decay in aging. Biochim. Biophys. Acta Mol. Basis Dis..

[5] Ames B., Liu J., Atamna H., Hagen T. (2003). Delaying the mitochondrial decay of aging in the brain. Clin. Neurosci. Res..

[6] Raz N., Lindenberger U., Rodrigue K., Kennedy K., Head D., Williamson A., Dahle C., Gerstorf D., Acker J. (2005). Regional brain changes in aging healthy adults: general trends, individual differences and modifiers. Cereb. Cortex.

[7] Lin M., Beal M. (2006). Mitochondrial dysfunction and oxidative stress in neurodegenerative diseases. Nature.

[8] Drevets W. (1999). Prefrontal cortical—amygdalar metabolism in major depression. Ann. N. Y. Acad. Sci..

[9] Prabakaran S., Swatton J., Ryan M., Huffaker S., Huang J., Griffin J., Wayland M., Freeman T., Dudbridge F., Lilley K. (2004). Mitochondrial dysfunction in schizophrenia: Evidence for compromised brain metabolism and oxidative stress. Mol. Psychiatry.

[10] Kato T., Kato N. (2000). Mitochondrial dysfunction in bipolar disorder. Bipolar Disord..

[11] Sieber F.E., Derrer S.A., Saudek C.D., Traystman R.J. (1989). Effect of hypoglycemia on cerebral metabolism and carbon dioxide responsivity. Am. J. Physiol..

[12] Sieber F.E., Traystman R.J. (1992). Special issues: Glucose and the brain. Crit. Care Med..

[13] Purves W.K., Sadava D., Orians G.H. (2004). Life: The Science of Biology: Plants and Animals.

[14] Lehninger A., Nelson D., Cox M. (2005). Lehninger Principles of Biochemistry.

[15] Wiesinger H., Hamprecht B., Dringen R. (1997). Metabolic pathways for glucose in astrocytes. Glia.

[16] Kauppinen R.A., McMahon H.T., Nicholls D.G. (1988). Ca^2+^-dependent and Ca^2+^-independent glutamate release, energy status and cytosolic free Ca^2+^ concentration in isolated nerve terminals following metabolic inhibition: Possible relevance to hyoglycaemia and anoxia. Neuroscience.

[17] Dringen R., Hamprecht B. (1992). Glucose, insulin, and insulin-like growth factor I regulate the glycogen content of astroglia-rich primary cultures. J. Neurochem..

[18] Hertz L., Yager J., Juurlink B. (1995). Astrocyte survival in the absence of exogenous substrate: Comparison of immature and mature cells. Int. J. Dev. Neurosci..

[19] Gibbs M.E., Anderson D.G., Hertz L. (2006). Inhibition of glycogenolysis in astrocytes interrupts memory consolidation in young chickens. Glia.

[20] Guyton A.C., Hall J.E. (1981). Textbook of Medical Physiology.

[21] Love R.J., Webb W.G., Kirshner H.S., Halliburton D.B., Gross P. (1996). Neurology for the Speech-Language Pathologist.

[22] McIlwain H. (1959). Thiols and the control of carbohydrate metabolism in cerebral tissues. Biochem. J..

[23] Gold P.E. (1986). Glucose modulation of memory storage processing. Behav. Neural Biol..

[24] Lee M.K., Graham S.N., Gold P.E. (1988). Memory enhancement with posttraining intraventricular glucose injections in rats. Behav. Neurosci..

[25] Kopf S.R., Baratti C.M. (1996). Effects of posttraining administration of glucose on retention of a habituation response in mice: Participation of a central cholinergic mechanism. Neurobiol. Learn. Mem..

[26] Hall J.L., Gonder-Frederick L., Chewning W., Silveira J., Gold P. (1989). Glucose enhancement of performance of memory tests in young and aged humans. Neuropsychologia.

[27] Gonder-Frederick L., Hall J., Vogt J., Cox D., Green J., Gold P. (1987). Memory enhancement in elderly humans: Effects of glucose ingestion. Physiol. Behav..

[28] Craft S., Murphy C., Wemstrom J. (1994). Glucose effects on complex memory and nonmemory tasks: The influence of age, sex, and glucoregulatory response. Psychobiology.

[29] Messier C., Gagnon M., Knott V. (1997). Effect of glucose and peripheral glucose regulation on memory in the elderly. Neurobiol. Aging.

[30] Craft S., Zallen G., Baker L.D. (1992). Glucose and memory in mild senile dementia of the Alzheimer type. J. Clin. Exp. Neuropsychol..

[31] Manning C.A., Ragozzino M.E., Gold P.E. (1993). Glucose enhancement of memory in patients with probable senile dementia of the Alzheimer’s type. Neurobiol. Aging.

[32] Manning C.A., Honn V.J., Stone W.S., Jane J.S., Gold P.E. (1998). Glucose effects on cognition in adults with Down’s syndrome. Neuropsychologia.

[33] Newcomer J.W., Craft S., Fucetola R., Moldin S.O., Selke G., Paras L., Miller R. (1999). Glucose-induced increase in memory performance in patients with schizophrenia. Schizophr. Bull..

[34] Fucetola R., Newcomer J.W., Craft S., Melson A.K. (1999). Age-and dose-dependent glucose-induced increases in memory and attention in schizophrenia. Psychiatry Res..

[35] Gradman T.J., Laws A., Thompson L.W., Reaven G.M. (1993). Verbal learning and/or memory improves with glycemic control in older subjects with non-insulin-dependent diabetes mellitus. J. Am. Geriatr. Soc..

[36] Meneilly G.S., Cheung E., Tessier D., Yakura C., Tuokko H. (1993). The effect of improved glycemic control on cognitive functions in the elderly patient with diabetes. J. Gerontol..

[37] Gold P.E., Vogt J.A., Hall J.L. (1986). Glucose effects on memory: Behavioral and pharmacological characteristics. Behav. Neural Biol..

[38] Sunram-Lea S., Owen L., Finnegan Y., Hu H. Dose-response investigation into glucose facilitation of memory performance and mood in healthy young adults. J. Psychopharmacol..

[39] Hoyland A., Lawton C., Dye L. (2008). Acute effects of macronutrient manipulations on cognitive test performance in healthy young adults: a systematic research review. Neurosci. Biobehav. Rev..

[40] Sünram-Lea S., Foster J., Durlach P., Perez C. (2001). Glucose facilitation of cognitive performance in healthy young adults: Examination of the influence of fast-duration, time of day and pre-consumption plasma glucose levels. Psychopharmacology.

[41] Kennedy D., Scholey A. (2000). Glucose administration, heart rate and cognitive performance: Effects of increasing mental effort. Psychopharmacology.

[42] Scholey A., Harper S., Kennedy D. (2001). Cognitive demand and blood glucose. Physiol. Behav..

[43] Sünram-Lea S., Foster J., Durlach P., Perez C. (2002). Investigation into the significance of task difficulty and divided allocation of resources on the glucose memory facilitation effect. Psychopharmacology.

[44] McNay E.C., Fries T.M., Gold P.E. (2000). Decreases in rat extracellular hippocampal glucose concentration associated with cognitive demand during a spatial task. Proc. Natl. Acad. Sci. USA.

[45] Scholey A.B., Harper S., Kennedy D.O. (2001). Cognitive demand and blood glucose. Physiol. Behav..

[46] Volpe B., Hirst W. (1983). Amnesia following the rupture and repair of an anterior communicating artery aneurysm. J. Neurol. Neurosurg. Psychiatry.

[47] Kuwert T., Hömberg V., Steinmetz H., Unverhau S., Langen K.J., Herzog H., Feinendegen L. (1993). Posthypoxic amnesia: Regional cerebral glucose consumption measured by positron emission tomography. J. Neurol. Sci..

[48] De la Torre J., Fortin T., Park G., Pappas B., Richard M. (1993). Brain blood flow restoration “rescues” chronically damaged rat CA1 neurons. Brain Res..

[49] Crowley J., Wesensten N., Kamimori G., Devine J., Iwanyk E., Balkin T. (1992). Effect of high terrestrial altitude and supplemental oxygen on human performance and mood. Aviat. Space Environ. Med..

[50] Weaver L., Hopkins R., Chan K., Churchill S., Elliott C., Clemmer T., Orme J., Thomas F., Morris A. (2002). Hyperbaric oxygen for acute carbon monoxide poisoning. N. Engl. J. Med..

[51] Weiskopf R., Feiner J., Hopf H., Viele M., Watson J., Kramer J., Ho R., Toy P. (2002). Oxygen reverses deficits of cognitive function and memory and increased heart rate induced by acute severe isovolemic anemia. Anesthesiology.

[52] Eustache F., Rioux P., Desgranges B., Marchal G., Petit-Taboué M.C., Dary M., Lechevalier B., Baron J.C. (1995). Healthy aging, memory subsystems and regional cerebral oxygen consumption. Neuropsychologia.

[53] Strehler B., Sarkander H.-I., Cervós-Navarro J. (1983). Fundamental mechanisms of neuronal aging. Brain Aging: Neuropathology and Neuropharmacology, Aging.

[54] Naritomi H., Meyer J.S., Sakai F., Yamaguchi F., Shaw T. (1979). Effects of advancing age on regional cerebral blood flow: Studies in normal subjects and subjects with risk factors for atherothrombotic stroke. Arch. Neurol..

[55] Davis S.M., Ackerman R.H., Correia J.A., Alpert N.M., Chang J., Buonanno F., Kelley R.E., Rosner B., Taveras J.M. (1983). Cerebral blood flow and cerebrovascular CO2 reactivity in stroke age normal controls. Neurology.

[56] Lassen N.A., Ingvar D.H., Amaducci L., Davison A.N., Antuono P. (1980). Blood flow studies in the aging normal brain and in senile dementia. Aging of the Brain and Dementia.

[57] Rowan J., McAlpine C., Matheson M., Patterson J. (1981). CBF, vasomotor tone, and intelligence rating in nonagenarians. J. Cereb. Blood Flow Metab..

[58] Edwards A., Hart G. (1974). Hyperbaric oxygenation and the cognitive functioning of the aged. J. Am. Geriatr. Soc..

[59] Raskin A., Gershon S., Crook T., Sathananthan G., Ferris S. (1978). The effects of hyperbaric and normobaric oxygen on cognitive impairment in the elderly. Arch. Gen. Psychiatry.

[60] Walker B.B., Sandman C.A. (1979). Human visual evoked responses are related to heart rate. J. Comp. Physiol. Psychol..

[61] Moss M., Scholey A., Wesnes K. (1998). Oxygen administration selectively enhances cognitive performance in healthy young adults: A placebo-controlled double-blind crossover study. Psychopharmacology.

[62] Scholey A., Moss M., Wesnes K. (1998). Oxygen and cognitive performance: The temporal relationship between hyperoxia and enhanced memory. Psychopharmacology.

[63] Moss M., Scholey A. (1996). Oxygen administration enhances memory formation in healthy young adults. Psychopharmacology.

[64] Scholey A., Moss M., Neave N., Wesnes K. (1999). Cognitive performance, hyperoxia, and heart rate following oxygen administration in healthy young adults. Physiol. Behav..

[65] Chung S., Lee H., Choi M., Tack G., Lee B., Yi J., Kim H., Lee B. (2008). A study on the effects of 40% oxygen on addition task performance in three levels of difficulty and physiological signals. Int. J. Neurosci..

[66] Winder R., Borrill J. (1998). Fuels for memory: The role of oxygen and glucose in memory enhancement. Psychopharmacology.

[67] Yerkes R.M., Dodson J.D. (1908). The relation of strength of stimulus to rapidity of habit formation. J. Comp. Neurol. Psychol..

[68] Gladden L. (2004). Lactate metabolism: A new paradigm for the third millennium. J. Physiol..

[69] Lee J., Kim Y., Koh J. (2001). Protection by pyruvate against transient forebrain ischemia in rats. J. Neurosci..

[70] Wieloch T. (1985). Hypoglycemia-induced neuronal damage prevented by an *N*-methyl-D-aspartate antagonist. Science.

[71] Ying W., Chen Y., Alano C., Swanson R. (2002). Tricarboxylic acid cycle substrates prevent PARP-mediated death of neurons and astrocytes. J. Cereb. Blood Flow Metab..

[72] Suh S., Aoyama K., Matsumori Y., Liu J., Swanson R. (2005). Pyruvate administered after severe hypoglycemia reduces neuronal death and cognitive impairment. Diabetes.

[73] Kirino T. (2000). Delayed neuronal death. Neuropathology.

[74] Yu Y.M., Kim J.B., Lee K.W., Kim S.Y., Han P.L., Lee J.K. (2005). Inhibition of the cerebral ischemic injury by ethyl pyruvate with a wide therapeutic window. Stroke.

[75] Ragozzino M.E., Hellems K., Lennartz R.C., Gold P.E. (1995). Pyruvate infusions into the septal area attenuate spontaneous alternation impairments induced by intraseptal morphine injections. Behav. Neurosci..

[76] Krebs D.L., Parent M.B. (2005). Hippocampal infusions of pyruvate reverse the memory-impairing effects of septal muscimol infusions. Eur. J. Pharmacol..

[77] Izumi Y., Zorumski C.F. (1997). Involvement of nitric oxide in low glucose-mediated inhibition of hippocampal long-term potentiation. Synapse.

[78] Izumi Y., Katsuki H., Zorumski C.F. (1997). Monocarboxylates (pyruvate and lactate) as alternative energy substrates for the induction of long-term potentiation in rat hippocampal slices. Neurosci. Lett..

[79] Hoyer S. (1993). Abnormalities in brain glucose utilization and its impact on cellular and molecular mechanisms in sporadic dementia of Alzheimer type. Ann. N. Y. Acad. Sci..

[80] Parnetti L., Gaiti A., Polidori M., Brunetti M., Palumbo B., Chionne F., Cadini D., Cecchetti R., Senin U. (1995). Increased cerebrospinal fluid pyruvate levels in Alzheimer’s disease. Neurosci. Lett..

[81] Pugliese M., Carrasco J., Andrade C., Mas E., Mascort J., Mahy N. (2005). Severe cognitive impairment correlates with higher cerebrospinal fluid levels of lactate and pyruvate in a canine model of senile dementia. Prog. Neuro-Psychopharmacol. Biol. Psychiatry.

[82] Balsom P., Soderlund K., Sjodin B., Ekblom B. (1995). Skeletal muscle metabolism during short duration high-intensity exercise: influence of creatine supplementation. Acta Physiol. Scand..

[83] Walker J. (1979). Creatine: Biosynthesis, regulation, and function (Chick embryo experiments, dietary aspects). Adv. Enzymol. Relat. Areas Mol. Biol..

[84] Snow R.J., Murphy R.M. (2003). Factors influencing creatine loading into human skeletal muscle. Exerc. Sport Sci. Rev..

[85] Kamber M., Koster M., Kreis R., Walker G., Boesch C., Hoppler H. (1999). Creatine supplementation—part I: Performance, clinical chemistry, and muscle volume. Med. Sci. Sports Exerc..

[86] Persky A.M., Brazeau G.A., Hochhaus G. (2003). Pharmacokinetics of the dietary supplement creatine. Clin. Pharmacokinet..

[87] Conway M., Clark J. (1996). Creatine and Creatine Phosphate: Scientific and Clinical Perspectives.

[88] Rango M., Castelli A., Scarlato G. (1997). Energetics of 3.5 s neural activation in humans: A 31P MR spectroscopy study. Magn. Reson. Med..

[89] Sappey-Marinier D., Calabrese G., Fein G., Hugg J., Biggins C., Weiner M. (1992). Effect of photic stimulation on human visual cortex lactate and phosphates using ^1^H and ^31^P magnetic resonance spectroscopy. J. Cereb. Blood Flow Metab..

[90] Dechent P., Pouwels P., Wilken B., Hanefeld F., Frahm J. (1999). Increase of total creatine in human brain after oral supplementation of creatine-monohydrate. Am. J. Physiol..

[91] Lyoo I., Kong S., Sung S., Hirashima F., Parow A., Hennen J., Cohen B., Renshaw P. (2003). Multinuclear magnetic resonance spectroscopy of high-energy phosphate metabolites in human brain following oral supplementation of creatine-monohydrate. Psychiatry Res. Neuroimaging.

[92] Jost C., Van der Zee C., Oerlemans F., Verheij M., Streijger F., Fransen J., Heerschap A., Cools A., Wieringa B. (2002). Creatine kinase B-driven energy transfer in the brain is important for habituation and spatial learning behaviour, mossy fibre field size and determination of seizure susceptibility. Eur. J. Neurosci..

[93] Wilken B., Ramirez J., Probst I., Richter D., Hanefeld F. (1998). Creatine protects the central respiratory network of mammals under anoxic conditions. Pediatr. Res..

[94] Balestrino M., Rebaudo R., Lunardi G. (1999). Exogenous creatine delays anoxic depolarization and protects from hypoxic damage: Dose-effect relationship. Brain Res..

[95] Holtzman D., Togliatti A., Khati I., Jensen F. (1998). Creatine increases survival and suppresses seizures in the hypoxic immature rat. Pediatr. Res..

[96] Sullivan P., Geiger J., Mattson M., Scheff S. (2000). Dietary supplement creatine protects against traumatic brain injury. Ann. Neurol..

[97] Shear D., Haik K., Dunbar G. (2000). Creatine reduces 3-nitropropionic-acid-induced cognitive and motor abnormalities in rats. NeuroReport.

[98] Wyss M., Braissant O., Pischel I., Salomons G.S., Schulze A., Stockler S., Wallimann T., Salomons G.S., Wyss M. (2007). Creatine and creatine kinase in health and disease—A bright future ahead?. Creatine and Creatine Kinase in Health and Disease.

[99] McMorris T., Harris R., Swain J., Corbett J., Collard K., Dyson R., Dye L., Hodgson C., Draper N. (2006). Effect of creatine supplementation and sleep deprivation, with mild exercise, on cognitive and psychomotor performance, mood state, and plasma concentrations of catecholamines and cortisol. Psychopharmacology.

[100] McMorris T., Harris R., Howard A., Langridge G., Hall B., Corbett J., Dicks M., Hodgson C. (2007). Creatine supplementation, sleep deprivation, cortisol, melatonin and behavior. Physiol. Behav..

[101] Watanabe A., Kato N., Kato T. (2002). Effects of creatine on mental fatigue and cerebral hemoglobin oxygenation. Neurosci. Res..

[102] Ling J., Kritikos M., Tiplady B. (2009). Cognitive effects of creatine ethyl ester supplementation. Behav. Pharmacol..

[103] Rawson E., Lieberman H., Walsh T., Zuber S., Harhart J., Matthews T. (2008). Creatine supplementation does not improve cognitive function in young adults. Physiol. Behav..

[104] Rae C., Digney A., McEwan S., Bates T. (2003). Oral creatine monohydrate supplementation improves brain performance: A double-blind, placebo-controlled, cross-over trial. Proc. R. Soc. B Biol. Sci..

[105] McMorris T., Mielcarz G., Harris R., Swain J., Howard A. (2007). Creatine supplementation and cognitive performance in elderly individuals. Neuropsychol. Dev. Cogn. B Aging Neuropsychol. Cogn..

[106] Laakso M., Hiltunen Y., Könönen M., Kivipelto M., Koivisto A., Hallikainen M., Soininen H. (2003). Decreased brain creatine levels in elderly apolipoprotein E 4 carriers. J. Neural Transm..

[107] Steiber A., Kerner J., Hoppel C. (2004). Carnitine: A nutritional, biosynthetic, and functional perspective. Mol. Aspects Med..

[108] Liedtke A., Nellis S., Whitesell L., Mahar C. (1982). Metabolic and mechanical effects using L- and D-carnitine in working swine hearts. Am. J. Physiol..

[109] Simon E. (2005). Fatty acid oxidation defects as a cause of neuromyopathic disease in infants and adults. Clin. Lab..

[110] Wasserman K., Whipp B. (1975). Excercise physiology in health and disease. Am. Rev. Respir. Dis..

[111] Mingrone G., Greco A., Capristo E., Benedetti G., Giancaterini A., Gaetano A., Gasbarrini G. (1999). L-Carnitine improves glucose disposal in type 2 diabetic patients. J. Am. Coll. Nutr..

[112] Nalecz K.A., Nalecz M.J. (1996). Carnitine—a known compound, a novel function in neural cells. Acta Neurobiol. Exp. (Wars.).

[113] Imperato A., Ramacci M., Angelucci L. (1989). Acetyl-L-carnitine enhances acetylcholine release in the striatum and hippocampus of awake freely moving rats. Neurosci. Lett..

[114] Furlong J. (1996). Acetyl-L-carnitine: Metabolism and applications in clinical practice. Altern. Med. Rev..

[115] Forloni G., Angeretti N., Smiroldo S. (1994). Neuroprotective activity of acetyl-L-carnitine: Studies *in vitro*. J. Neurosci. Res..

[116] Ishii T., Shimpo Y., Matsuoka Y., Kinoshita K. (2000). Anti-apoptotic effect of acetyl-L-carnitine and L-carnitine in primary cultured neurons. Jpn. J. Pharmacol..

[117] Binienda Z.K. (2003). Neuroprotective effects of L-carnitine in induced mitochondrial dysfunction. Ann. N. Y. Acad. Sci..

[118] Ghirardi O., Vertechi M., Vesci L., Canta A., Nicolini G., Galbiati S., Ciogli C., Quattrini G., Pisanto C., Cundari S., Rigamonti L.M. (2005). Chemotherapy-induced allodinia: Neuroprotective effect of acetyl-L-carnitine. In Vivo.

[119] Lolic M.M., Fiskum G., Rosenthal R.E. (1997). Neuroprotective effects of acetyl-L-carnitine after stroke in rats. Ann. Emerg. Med..

[120] Rosenthal R.E., Williams R., Bogaert Y.E., Getson P.R., Fiskum G. (1992). Prevention of postischemic canine neurological injury through potentiation of brain energy metabolism by acetyl-L-carnitine. Stroke.

[121] Parnetti L., Gaiti A., Mecocci P., Cadini D., Senin U. (1992). Pharmacokinetics of IV and oral acetyl-L-carnitine in a multiple dose regimen in patients with senile dementia of Alzheimer type. Eur. J. Clin. Pharmacol..

[122] Montgomery S., Thal L., Amrein R. (2003). Meta-analysis of double blind randomized controlled clinical trials of acetyl-L-carnitine *versus* placebo in the treatment of mild cognitive impairment and mild Alzheimer’s disease. Int. Clin. Psychopharmacol..

[123] Malaguarnera M., Cammalleri L., Gargante M., Vacante M., Colonna V., Motta M. (2007). L-Carnitine treatment reduces severity of physical and mental fatigue and increases cognitive functions in centenarians: A randomized and controlled clinical trial. Am. J. Clin. Nutr..

[124] Junien C., Nathanielsz P. (2007). Report on the IASO Stock Conference 2006: Early and lifelong environmental epigenomic programming of metabolic syndrome, obesity and type II diabetes. Obes. Rev..

[125] Fraga M.F., Ballestar E., Paz M.F., Ropero S., Setien F., Ballestar M.L., Heine-Suñer D., Cigudosa J.C., Urioste M., Benitez J. (2005). Epigenetic differences arise during the lifetime of monozygotic twins. Proc. Natl. Acad. Sci. USA.

[126] Fraga M.F., Esteller M. (2007). Epigenetics and aging: the targets and the marks. Trends Genet..

[127] Christensen B.C., Houseman E.A., Marsit C.J., Zheng S., Wrensch M.R., Wiemels J.L., Nelson H.H., Karagas M.R., Padbury J.F., Bueno R. (2009). Aging and environmental exposures alter tissue-specific DNA methylation dependent upon CpG island context. PLoS Genet..

[128] Lomba A., Milagro F.I., García-Díaz D.F., Marti A., Campión J., Martínez J.A. (2010). Obesity induced by a pair-fed high fat sucrose diet: Methylation and expression pattern of genes related to energy homeostasis. Lipids Health Dis..

[129] Whittle J.R., Powell M.J., Popov V.M., Shirley L.A., Wang C., Pestell R.G. (2007). Sirtuins, nuclear hormone receptor acetylation and transcriptional regulation. Trends Endocrinol. Metab..

